# Local Atrophy Observed in Subjective Cognitive Decline Varies Based on Questionnaire Employed in ADNI

**DOI:** 10.1093/geroni/igab046.2414

**Published:** 2021-12-17

**Authors:** Cassandra Morrison, Mahsa Dadar, Neda shafiee, Louis Collins

**Affiliations:** 1 McConnell Brain Imaging Centre, Montreal Neurological Institute, McGill University, Montréal, Quebec, Canada; 2 McGill University, Montreal, Quebec, Canada; 3 McConnell Brain Imaging Centre, Montreal Neurological Institute, McGill University, Montreal, Quebec, Canada

## Abstract

Background: Subjective cognitive decline (SCD) may be associated with increased risk for Alzheimer’s disease. However, neither research nor clinical practices have implemented a universal approach to operationalize SCD. This study was designed to determine whether four different methods of defining SCD influence atrophy differences observed between SCD and normal controls (NC). Methods: We included MRI scans from 273 participants (NC and SCD) from the Alzheimer’s Disease Neuroimaging Initiative. We used four methods to operationalize SCD: Cognitive Change Index (CCI), Everyday Cognition Scale (ECog), Worry, and ECog+Worry. Deformation-based morphometry was performed to examine volumetric change at the lateral ventricles, amygdala, and superior temporal regions (CerebrA atlas; Manera et al., 2020)). A previously validated MRI analysis method (SNIPE) was used for volume and grading of the hippocampus and entorhinal cortex (Coupe et al., 2012). A logistic regression was completed to examine the association between diagnosis and atrophy in SCD and NC. Results: Left hippocampal grading was lower in SCD than NC with the CCI (p=.041) and Worry (p=.021). When using ECog+Worry, smaller left entorhinal volume was observed in SCD than NC (p=.025). Both the right (p=.008) and left (p=.003) superior temporal regions were smaller in SCD than NC, with only the ECog. Conclusion: Although SCD questionnaires are designed to measure the same construct, the results here suggest otherwise. These results suggest that the SCD questionnaire employed will influence whether atrophy is observed in SCD relative to NC. Future research is warranted to better understand how different methodologies result in inconsistent findings.

